# Interobserver variability in assessing preoperative imaging biomarkers for cerebellar mutism syndrome: a multiobserver pilot study

**DOI:** 10.1007/s00247-025-06326-y

**Published:** 2025-07-17

**Authors:** Aske Foldbjerg Laustsen, Rob Dineen, Jurgita Ilginiene, Jonathan Kjær Grønbæk, Astrid Marie Sehested, Kjeld Schmiegelow, René Mathiasen, Marianne Juhler, Shivaram Avula

**Affiliations:** 1 Department of Neurosurgery, Rigshospitalet, Blegdamsvej 9, Copenhagen, 2100 Denmark; 2 Department of Paediatrics and Adolescent Medicine, Rigshospitalet, Copenhagen, Denmark; 3 Department of Radiology, Nottingham University Hospitals NHS Trust, Nottingham, United Kingdom; 4 Department of Radiology, Rigshospitalet, Copenhagen, Denmark; 5 Department of Neurosurgery, Aarhus University Hospital, Aarhus, Denmark; 6 Department of Radiology, Alder Hey Children′s NHS Foundation Trust, Liverpool, United Kingdom

**Keywords:** Cerebellar mutism syndrome, Neuroimaging, Observer variation, Posterior fossa syndrome

## Abstract

**Background:**

Cerebellar mutism syndrome is a well-known complication of paediatric posterior fossa tumour surgery. In recent years, several imaging biomarkers have been suggested to predict cerebellar mutism syndrome based on its probable pathoanatomical causes.

**Objective:**

This study investigates the reliability of preoperative imaging biomarkers for cerebellar mutism syndrome in paediatric posterior fossa tumours. Specifically, it examines the interobserver agreement on the size, invasion, and compression of selected regions of interest with structured magnetic resonance imaging (MRI) reporting.

**Materials and methods:**

Preoperative brain MRI scans from ten paediatric patients with posterior fossa tumours, conducted at a single institution, were analysed. The scans were evaluated by three neuroradiologists from three different institutions across two countries using a structured reporting format. The interobserver agreement was assessed using intraclass correlation coefficient and Fleiss’ kappa. All estimates were reported with a 95% confidence interval.

**Results:**

The study found good to excellent agreement in measuring tumour size, tumour volume, and the Evans index. Substantial agreement was found in tumour pathology and location. However, the interobserver agreement was unreliable for invasion and compression of the included anatomical structures.

**Conclusion:**

Findings from this study challenge the reliability of preoperative imaging biomarkers for cerebellar mutism syndrome, emphasising the need for further investigation into consistent and reproducible biomarkers relevant to this syndrome.

**Graphical Abstract:**

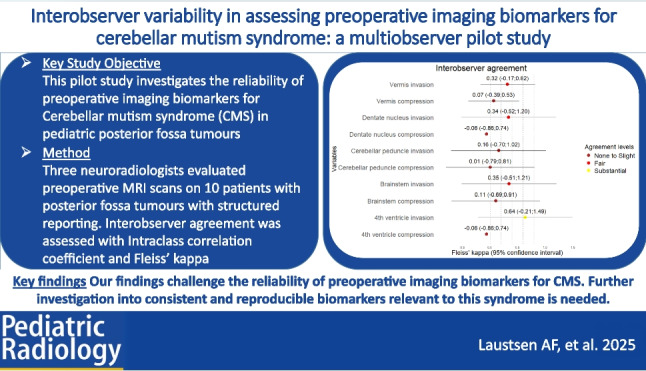

**Supplementary Information:**

The online version contains supplementary material available at 10.1007/s00247-025-06326-y.

## Introduction

Cerebellar mutism syndrome is a well-described occurrence following paediatric neuro-oncological posterior fossa surgery. The syndrome comprises speech impairment, emotional disturbances, ataxia, and hypotonia accompanied by brainstem signs [1]. The reported incidence varies between approximately 10–30% [2–8]. Risk factors include lower age, tumour located in the brainstem or midline, histopathological diagnosis of medulloblastoma, tumour invasion of the superior cerebellar peduncles, and larger tumour volume [2, 4, 8–10]. The syndrome has puzzled neurosurgeons and paediatric neurooncologists for years, as it seldom occurs in the adult population operated for a posterior fossa tumour. Current understanding attributes the syndrome to the disruption of the cerebellar-cerebral outflow tract – the dentato-thalamo-cortical pathway – resulting in cerebello-cerebral diaschisis [11].

Due to this probable pathoanatomical cause of the syndrome, various predictive models involving a combination of clinical patient characteristics and neuroradiographic biomarkers have been suggested over the years. In 2018, Liu et al. defined the Nottingham-Liverpool risk prediction model of postoperative cerebellar mutism syndrome, mainly relying on neuroradiographic features indicating the importance of invasion and compression of certain anatomical structures [12]. In 2019, Zhang et al. introduced the D(sag) and d(sag) as predictive factors for postoperative cerebellar mutism syndrome on a retrospective cohort [13]. D(sag) is the length over which the tumour invades or compresses the brainstem, whereas d(sag) represents the depth of tumour invasion or compression on the brainstem. The Rotterdam prediction model for postoperative cerebellar mutism syndrome was published later that same year, evaluating the results from Liu et al. and Zhang et al., and extending both models into the Rotterdam model, which involved more comprehensive radiological features indicative of anatomical invasion and compression [14]. Recently, Sidpra et al. published an improved prediction model based on an artificial neural network model, which included further comprehensive radiological features of tumour characteristics, anatomical invasion and compression, as well as age, and sex [15].

However, interobserver variability among radiologists remains a challenge in medical imaging, impacting the validity of prediction models. Ensuring the reproducibility of magnetic resonance imaging (MRI) variables is essential for these models to be reliable. Furthermore, increasing the granularity of the assessment criteria can improve the prediction of cerebellar mutism syndrome risk and severity.

To address this challenge, our pilot study aims to evaluate the interobserver variability among three neuroradiologists in assessing neuroradiographic features on MRI scans of children with posterior fossa brain tumours using a structured reporting format. We seek to identify the most consistent qualitative imaging findings for larger cohort assessments by refining the qualitative scores through a ranking system for specific criteria rather than a binary response. The increased granularity is designed to improve the correlation between imaging scores and the severity of cerebellar mutism syndrome, thereby validating the reliability of preoperative neuroradiographic biomarkers in cerebellar mutism syndrome risk and severity prediction.

## Materials and methods

We enrolled the first ten Danish paediatric patients operated for a posterior fossa tumour included in the European cerebellar mutism syndrome study (H-6–2014-002) into this pilot study. The study protocol for the European cerebellar mutism syndrome study is previously published [16]. All patients were affiliated with a single centre in Denmark and gave written consent. Preoperative routine clinical brain MRI scans on a 1.5-T (T) or 3-T scanner using a standardised diagnostic MRI protocol for paediatric brain tumours were examined for this study (Table 1).

**Table 1:** Patient demographics and magnetic field strength

Patient number	Age at diagnostic scan (years and months)	Sex	Magnetic field strength of diagnostic scan (tesla)	Available sequences
1	1y 2 m	Female	1.5	Ax Dual Echo, DWI, Cor FLAIR, Sag 3D T1 + C
2	7y 4 m	Male	1.5	Ax T2, T1, DWI, 3D T1 + C, Cor FLAIR
3	9y 3 m	Male	1.5	Ax T2, DWI, Cor FLAIR, Sag T1, 3D T1 + C
4	4y 7 m	Male	3.0	Ax T2, DWI, Cor FLAIR, Sag 3D T1, T1 + C
5	2y 4 m	Male	3.0	Ax T2, DWI, Cor T2, Sag 3D FLAIR, 3D T1, 3D T1 + C
6	4y 2 m	Female	3.0	Ax T2, DWI, SWI, Cor 3D FLAIR, Sag 3D T1, 3D T1 + C
7	6y 7 m	Female	3.0	Ax T2, DWI, Cor FLAIR, Sag T1, 3D T1 + C
8	15y 10 m	Male	1.5	Ax T2, DWI, T2*, Cor FLAIR, Sag T1, 3D T1 + C
9	4y 9 m	Male	3.0	Ax T2, DWI, T1, FLAIR, Sag 3D T1 + C
10	4y 4 m	Male	1.5	Ax T2, DWI, Cor FLAIR, Sag T1, 3D T1 + C

Ax axial, C contrast, Cor coronal, DWI diffusion-weighted imaging, FLAIR fluid-attenuated inversion recovery, Sag sagittal, T2* T2-star, 3D three-dimensional

**Fig. 1:**
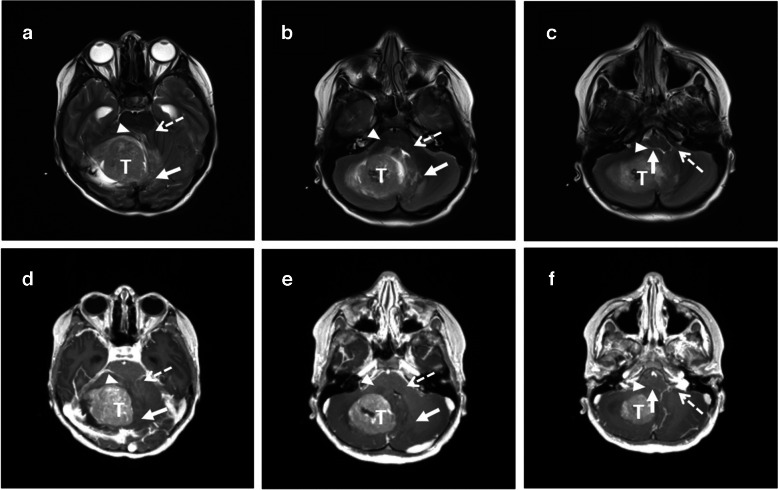
Axial T2 (**a**–**c**) and post-contrast T1 (**d**–**f**) magnetic resonance images in a 6-year-old girl (patient 7) with a posterior fossa tumour. Slices have been obtained at the level of the superior (**a**, **d**), middle (**b**,** e**), and inferior (**c**,** f**) cerebellar peduncles. **a**,** d** Compression of the vermis has a score of 4 (*arrow*), of the right superior cerebellar peduncle a score of 4 (*arrowhead*), and of the left superior cerebellar peduncle a score of 2 (*broken arrow*). **b**,** e** Compression of the region of the left dentate nucleus has a score of 3 (*arrow*), of the right middle cerebellar peduncle a score of 4 (*arrowhead*), and of the left middle cerebellar peduncle a score of 2 (*broken arrow*). **c**, **f** Compression of the medulla oblongata has a score of 3 (*arrow*), of the right inferior cerebellar peduncle a score of 3 (*arrowhead*), and of the left inferior cerebellar peduncle a score of 2 (*broken arrow*)*.* Scoring references were provided by the most experienced radiologist (R.D.). *T* posterior fossa tumour

A structured report including neuroradiographic features and regions of interest (ROI) was constructed (Table 2 and Supplementary Material 1). Three neuroradiologists evaluated ROI on each scan and documented findings in the structured report. The neuroradiologists’ experience in paediatric neuroradiology was 2 years (J.I.), 15 years (S.A.), and 17 years (R.D.), respectively. Scoring of compression is exemplified in Fig. 1.

**Table 2:** Neuroradiographic biomarkers and features

Variable	Definition	Outcome
Tumour characteristics		
Cranio-caudal	Maximal CC diameter of the tumour	Millimetres
Anterior–posterior	Maximal AP diameter of the tumour	Millimetres
Side-to-side	Maximal SS diameter of the tumour	Millimetres
Tumour size	$CC+AP+SS$	Millimetres
Tumour volume	$\frac{CC\ast AP\ast SS}2$	Cubic millimetres
Cystic appearance	More than 50% of the tumour	1) Yes 2) No 3) Equivocal or not available or suboptimal scan
Haemorrhage in tumour	Presence of haematoma in or surrounding the tumour	1. Yes 2. No 3. Equivocal or not available or suboptimal scan
Pathology	Neuroradiographic tumour diagnosis	1) Pilocytic astrocytoma 2) Medulloblastoma 3) Ependymoma 4) Atypical teratoid/rhabdoid tumour 5) Other
Location	Neuroradiographic location of the tumour	1) Vermis 2) Right cerebellar hemisphere 3) Left cerebellar hemisphere 4) 4th ventricle 5) Foramen Luschka 6) Cerebello-pontine angle 7) Medullary cistern 8) Mesencephalon 9) Pons 10) Medulla oblongata 11) Multifocal
Compressed anatomical structures		
D(sag)	D(sag) is the length over which the tumour invades the brainstem	Millimetres
d(sag)	d(sag) is the depth of invasion or compression on the brainstem	Millimetres
Size of pons	Cranio-caudal size of pons	Millimetres
Size of medulla oblongata	Cranio-caudal size of medulla oblongata	Millimetres
Size of lower brainstem	Size of pons + size of medulla oblongata	Millimetres
Tumour extension into the cerebral aqueduct	Tumour extending into the cerebral aqueduct	1) Yes 2) No 3) Equivocal or not available or suboptimal scan
Is the prepontine cistern obliterated?	The prepontine cistern visible on the scan	1) Yes 2) No 3) Equivocal or not available or suboptimal scan
Signs of intracranial dissemination	Signs of intracranial dissemination of the tumour	1) Yes 2) No 3) Equivocal or not available or suboptimal scan
Signs of spinal dissemination	Signs of spinal dissemination of the tumour	1) Yes 2) No 3) No spinal MRI 4) Equivocal or not available or suboptimal scan
Signs of hydrocephalus	Signs of hydrocephalus	1) No 2) Mild 3) Moderate 4) Severe 5) Equivocal or not available or suboptimal scan
Evans A	Maximal width of the frontal horns	Millimetres
Evans B	Maximal inner skull diameter	Millimetres
Evans index	Evans A/Evans B	Millimetres
Signs of transependymal periventricular oedema	Oedema in the periventricular transependymal zone	1) Yes 2) No 3) Equivocal or not available or suboptimal scan
Affected nucleus olivaris	Signs of affected inferior olivary nucleus	1) Yes 2) No 3) Equivocal or not available or suboptimal scan
Structural compression and invasion		
Compression of anatomical structures	a) Mass effect arising from tumour b) Structure adjacent to tumour, or if not adjacent to tumour, the intervening structure(s) is also compressed c) Displacement with significant distortion of shape and normal anatomy of the structure d) Displacement alone with maintenance of shape or normal anatomy alone is not sufficient	1) None 2) Uncertain 3) Displacement without morphological change 4) Mild compression (displacement and shape change) 5) Moderate to severe compression (displacement and shape change and distortion of adjacent anatomical structure) 6) Equivocal or not available or suboptimal scan
Invasion of anatomical structures except 4th ventricle	Invasion is present if there is a blurred margin between tumour and structure on more than 2 slices or in 2 different planes	1) None 2) Invasion uncertain and not convincing on available sequences 3) Only blurring of margins 4) Clear penetration of tumour into the structure or tumour arising from the structure 5) Equivocal or not available or suboptimal scan
Invasion of 4th ventricle		1) None 2) Invasion uncertain and not convincing on available sequences 3) Clear penetration of tumour into the structure or tumour arising from the structure 4) Equivocal or not available or suboptimal scan

*AP* anterior–posterior,* CC* cranio-caudal, *SS* side-to-side

### Statistics

To evaluate the agreement between the three raters, intraclass correlation coefficient (ICC) was used for continuous variables and Fleiss’ kappa was used for categorical variables [17–22]. ICC was reported as a “two-way” analysis with “agreement” for calculating absolute agreement between raters. ICC “consistency” was not used for the primary analysis but was included in Supplementary Material 3.

All estimates were reported with a 95% confidence interval (CI) [23]. If no disagreement was present, no CI could be reported.

A sensitivity analysis was performed on interobserver agreement among the two most experienced observers and compared with the overall results of the three observers. Continuous variables were calculated with ICC, and categorical variables were calculated with Cohen’s kappa. All estimates were reported with a 95% CI.

Statistical analyses were performed using RStudio (v. 4.3.1).

### Agreement limits

ICC: < 0.5 poor agreement, 0.5–0.75 moderate agreement, 0.75–0.9 good agreement, > 0.9 excellent agreement.

Fleiss’ kappa and Cohen’s kappa: < 0 no agreement, 0.01–0.2 none to slight agreement, 0.21–0.4 fair agreement, 0.41–0.6 moderate agreement, 0.61–0.8 substantial agreement, 0.81–100 almost perfect agreement [24].

## Results

**Fig. 2:**
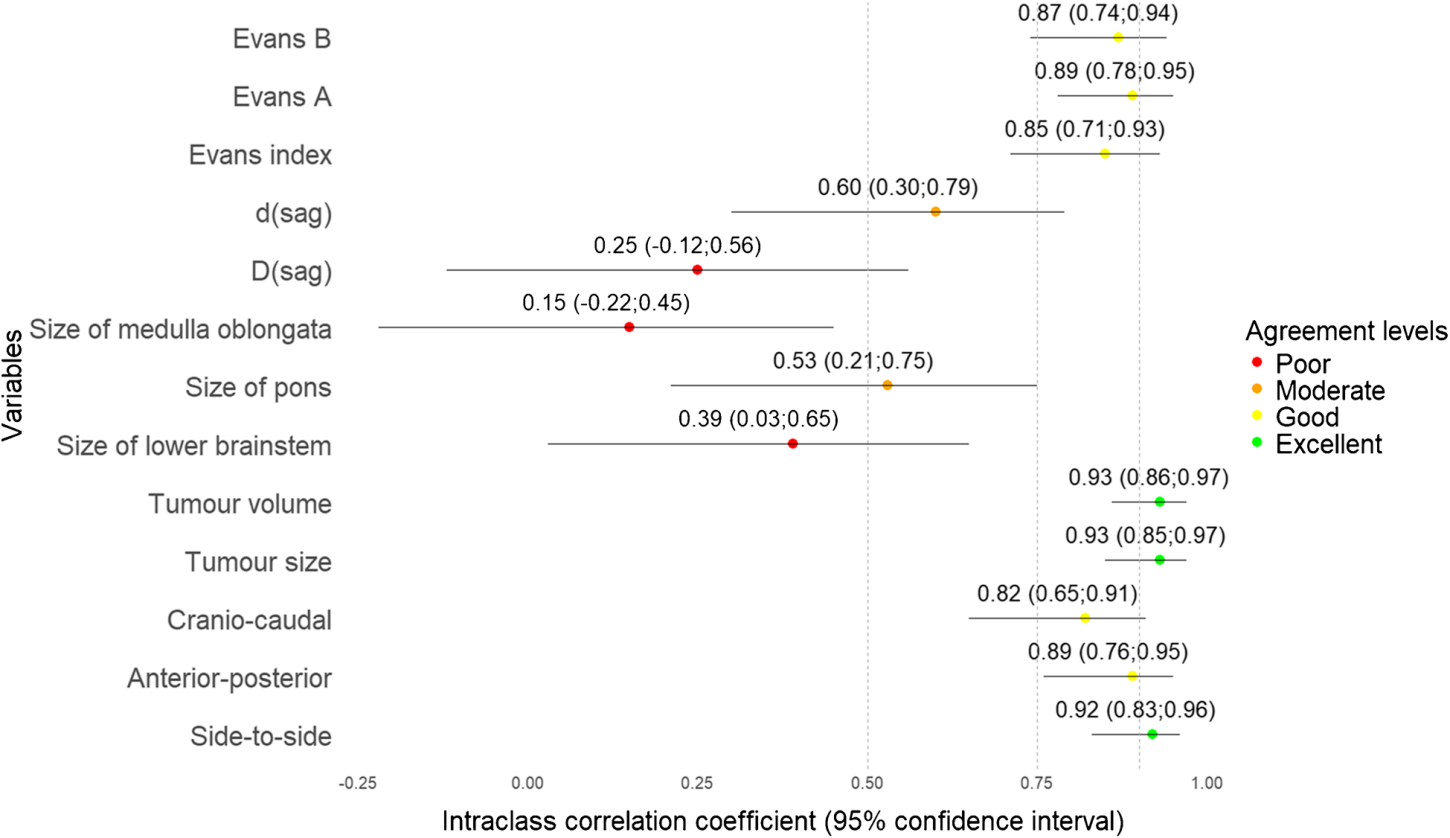
The forest plot for continuous variable agreement shows good to excellent agreement for side-to-side, anterior–posterior, cranio-caudal, tumour volume and size, Evans index, Evans A, and Evans B; moderate agreement for pons size and d(sag) (lower bound of confidence interval below 0.5 indicating uncertainty); and poor agreement for the remaining continuous biomarkers

**Fig. 3:**
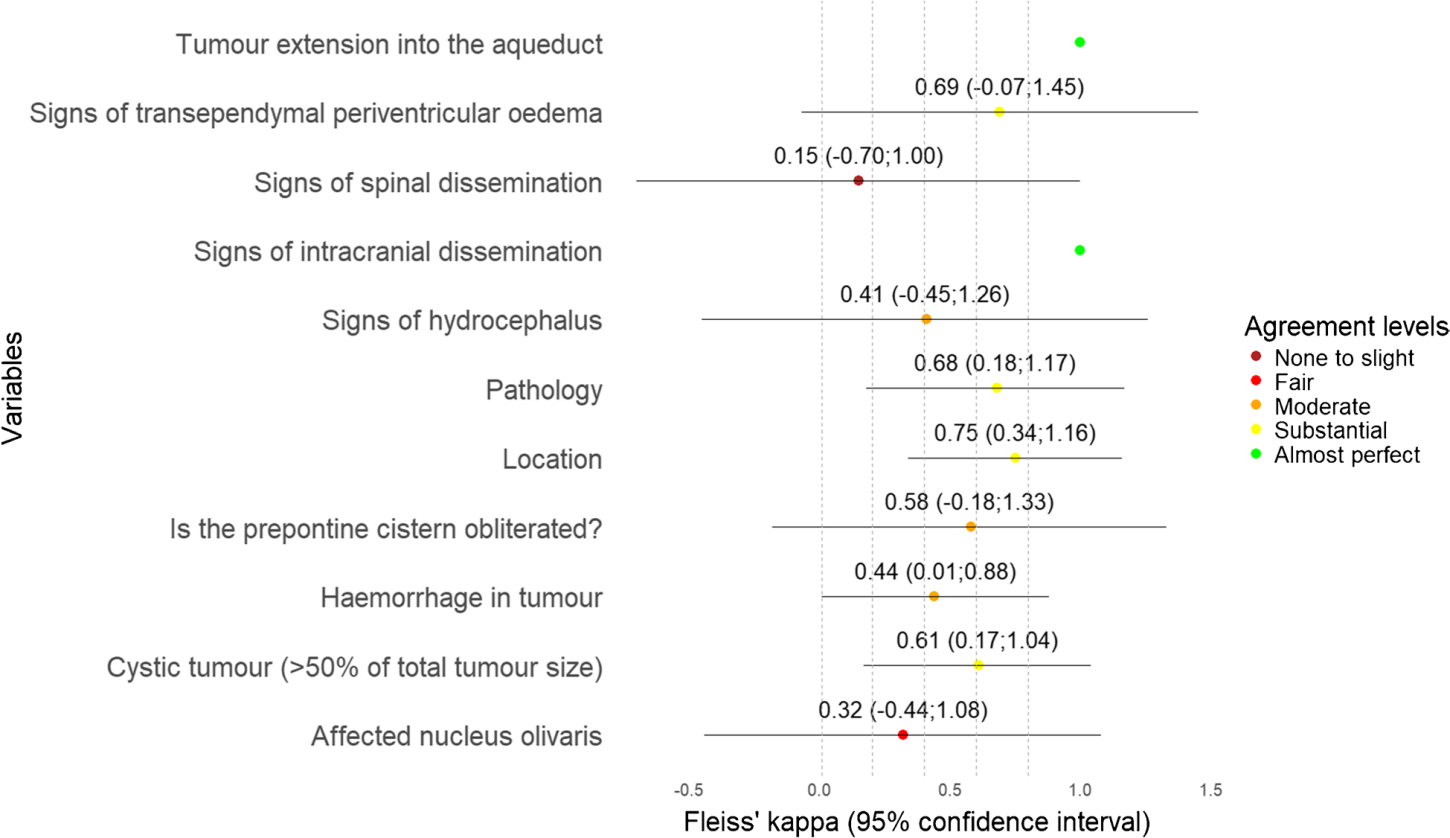
The forest plot for categorical variable agreement (except invasion and compression) shows fair to substantial agreement for tumour location, cystic tumour > 50% of total tumour size, haemorrhage in tumour, and tumour pathology. The remaining categorical variables vary from “none to slight” to substantial agreement, but have lower bound of the confidence interval below 0 indicating uncertainty to the agreement estimates

**Fig. 4:**
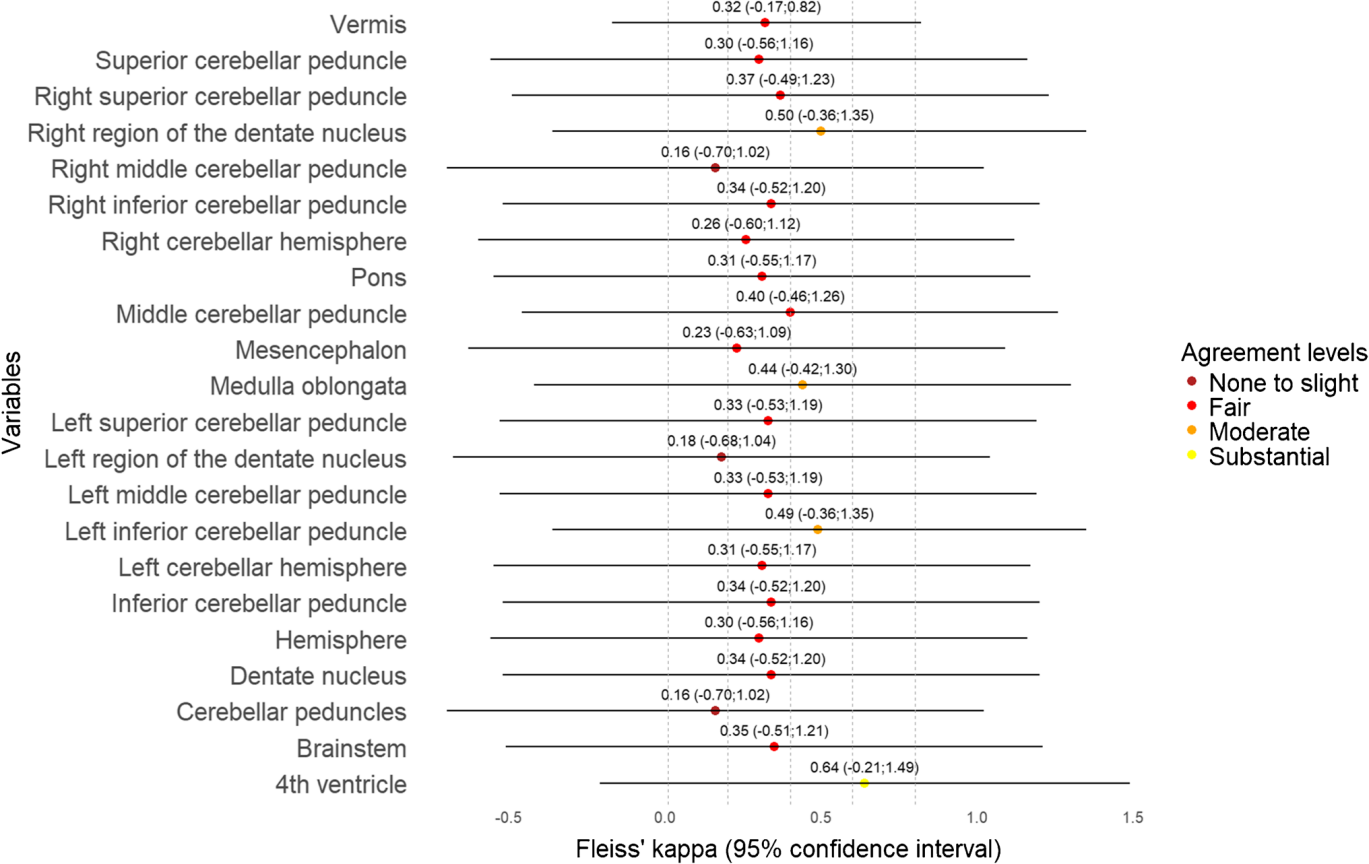
The forest plot for invasion agreement shows fair agreement for the majority of anatomical structures. The lower bounds of the confidence intervals fall below 0 for all Fleiss’ kappa estimates, indicating uncertainty in the agreement

**Fig. 5:**
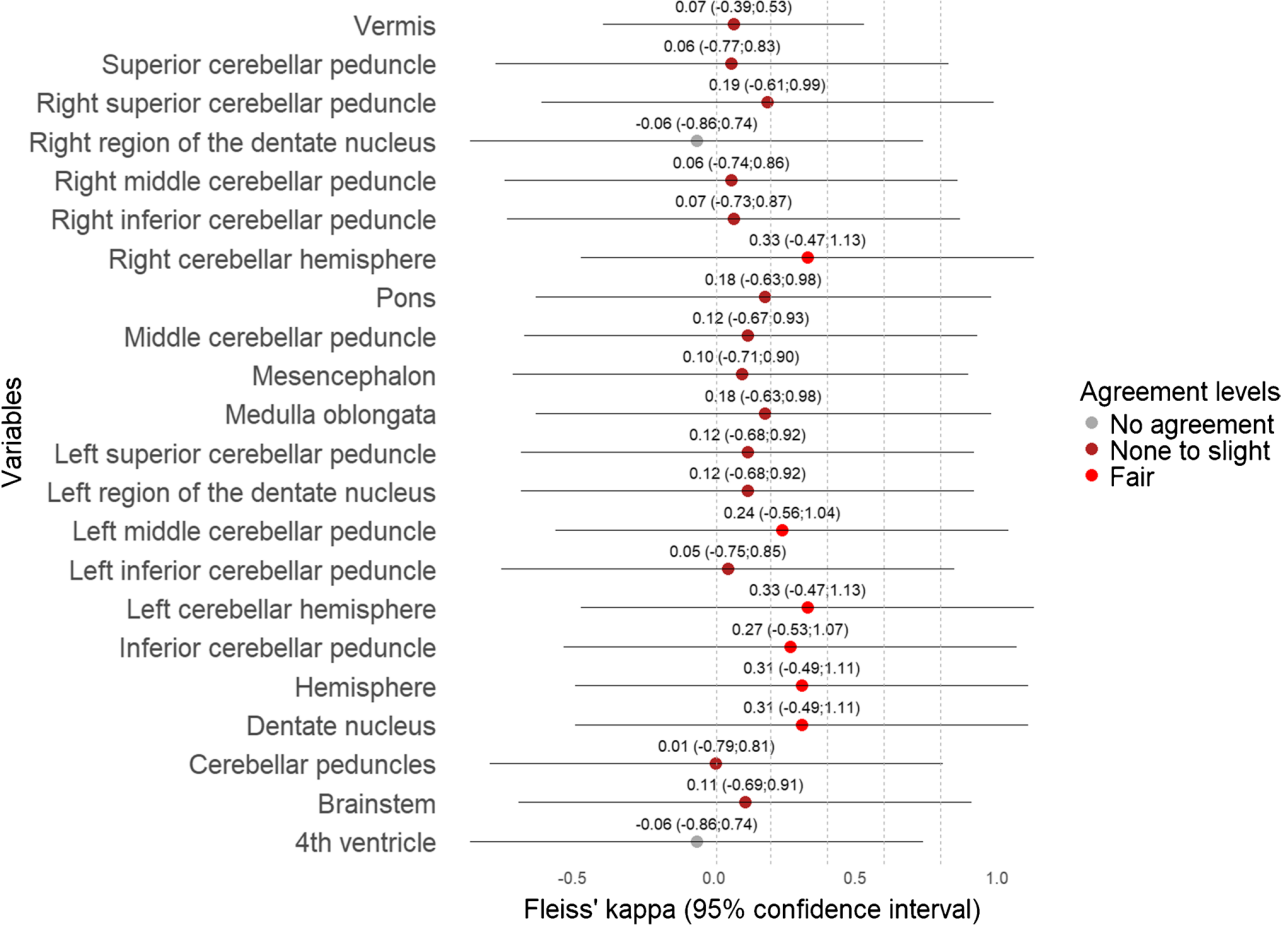
The forest plot for compression agreement shows none to slight agreement for the majority of anatomical structures. The lower bounds of the confidence intervals fall below 0 across all Fleiss’ kappa estimates, indicating uncertainty in the agreement

The ICC values among continuous variables showed that tumour size, including anterior–posterior, cranio-caudal, side-to-side, tumour volume, size of pons, Evans A, Evans B, Evans index, and d(sag) varied the least, ranging from moderate to excellent (Table 3 and Fig. 2). Among categorical variables, tumour location showed substantial agreement, ranging from fair to excellent, thus being the only reliable categorical imaging biomarker. Pathology (radiological diagnosis), cystic tumour > 50%, and intratumoural haemorrhage also had substantial agreement but ranged from “none to slight” to excellent (Table 4 and Fig. 3). In the case of invasion and compression (exemplified in Supplementary Material 1 and 2) of ROI (Table 5), agreement levels in all variables were unreliable, but invasion parameters (Fig. 4) tended to have higher agreement than compression (Fig. 5).

**Table 3:** Agreement for continuous variables

	ICC agreement (95% CI)
Evans B	0.87 (0.74; 0.94)
Evans A	0.89 (0.78; 0.95)
Evans index	0.85 (0.71; 0.93)
d(sag)	0.60 (0.30; 0.79)
D(sag)	0.25 (−0.12; 0.56)
Size of medulla oblongata	0.15 (−0.22; 0.45)
Size of pons	0.53 (0.21; 0.75)
Size of lower brainstem	0.39 (0.03; 0.65)
Tumour volume	0.93 (0.86; 0.97)
Tumour size	0.93 (0.85; 0.97)
Cranio-caudal size	0.82 (0.65; 0.91)
Anterior–posterior	0.89 (0.76; 0.95)
Side-to-side	0.92 (0.83; 0.96)

*CI *confidence interval, *ICC* intraclass correlation coefficient

**Table 4:** Agreement for categorical variables (except invasion and compression)

	Fleiss’ kappa (95% CI)
Tumour extension into the aqueduct	1^a^
Signs of transependymal periventricular oedema	0.69 (−0.07; 1.45)
Signs of spinal dissemination	0.15 (−0.70; 1.00)
Signs of intracranial dissemination	1^a^
Signs of hydrocephalus	0.41 (−0.45; 1.26)
Pathology	0.68 (0.18; 1.17)
Location	0.75 (0.34; 1.16)
Is the prepontine cistern obliterated?	0.58 (−0.18; 1.33)
Haemorrhage in tumour	0.44 (0.01; 0.88)
Cystic tumour (> 50% of total tumour size)	0.61 (0.17; 1.04)
Affected nucleus olivaris	0.32 (−0.44; 1.08)

^a^No observed disagreement – event did not occur. *CI* confidence interval

**Table 5:** Agreement for invasion and compression of anatomical structure

	Fleiss’ kappa (95% CI)	
	Invasion	Compression
Vermis	0.32 (−0.17; 0.82)	0.07 (−0.39; 0.53)
Superior cerebellar peduncle	0.30 (−0.56; 1.16)	0.06 (−0.77; 0.83)
Right superior cerebellar peduncle	0.37 (−0.49; 1.23)	0.19 (−0.61; 0.99)
Right region of the dentate nucleus	0.50 (−0.36; 1.35)	0.12 (−0.68; 0.92)
Right middle cerebellar peduncle	0.16 (−0.70; 1.02)	0.24 (−0.56; 1.04)
Right inferior cerebellar peduncle	0.34 (−0.52; 1.20)	0.05 (−0.75; 0.85)
Right cerebellar hemisphere	0.26 (−0.60; 1.12)	0.33 (−0.47; 1.13)
Pons	0.31 (−0.55; 1.17)	0.18 (−0.63; 0.98)
Middle cerebellar peduncle	0.23 (−0.62; 1.09)	0.06 (−0.74; 0.86)
Mesencephalon	0.23 (−0.63; 1.09)	0.10 (−0.71; 0.90)
Medulla oblongata	0.44 (−0.42; 1.30)	0.18 (−0.63; 0.98)
Left superior cerebellar peduncle	0.33 (−0.53; 1.19)	0.12 (−0.68; 0.92)
Left region of the dentate nucleus	0.18 (−0.68; 1.04)	0.12 (−0.67; 0.93)
Left middle cerebellar peduncle	0.40 (−0.46; 1.26)	0.27 (−0.53; 1.07)
Left inferior cerebellar peduncle	0.49 (−0.36; 1.35)	0.31 (−0.49; 1.11)
Left cerebellar hemisphere	0.31 (−0.55; 1.17)	0.33 (−0.47; 1.13)
Inferior cerebellar peduncle	0.34 (−0.52; 1.20)	0.07 (−0.73; 0.87)
Hemisphere	0.30 (−0.56; 1.16)	0.31 (−0.49; 1.11)
Dentate nucleus	0.34 (−0.52; 1.20)	−0.06 (−0.86; 0.74)
Cerebellar peduncles	0.16 (−0.70; 1.02)	0.007 (−0.79; 0.81)
Brainstem	0.35 (−0.51; 1.21)	0.11 (−0.69; 0.91)
4th ventricle	0.64 (−0.21; 1.49)	−0.06 (−0.86; 0.74)

*CI *confidence interval

Considering CIs, tumour size, tumour volume, tumour location, Evans A, Evans B, and Evans index were reliable imaging biomarkers. Furthermore, since we included 69 variables in this study, it is likely that some of the findings are due to chance (statistically, 3–4 of our agreement results).

The sensitivity analysis with the two most experienced observers gave an overall impression of right skewing of the ICC agreement values, whereas the agreement on categorical variables was scattered in both directions. As expected, 95% CI generally widened (Supplementary Material 3).

## Discussion

Our results question whether the included imaging biomarkers on preoperative brain MRI in this pilot study are sufficiently reliable for assessing the risk and severity of cerebellar mutism syndrome.

Despite the seemingly clear neuroanatomical definitions of the neuroradiographic biomarkers, it is crucial to recognise subjectivity in MRI evaluations. The interpretation of MRI findings in the context of paediatric brain tumours varies among radiologists due to differences in training, experience, and perceptual factors, which may reflect on the risk assessment of cerebellar mutism syndrome. Applying predictive models should consider MRI interpretation variabilities; standardised criteria for substrate evaluation, and use multiple reviewers to mitigate subjective biases [12, 14, 15].

Most studies rely on two observers to assess interobserver agreement. By incorporating a third observer, our study better reflects real-world clinical variability, and the inclusion of neuroradiologists from two countries provides insight into regional diagnostic differences. The findings of our sensitivity analysis comparing results from the two most experienced observers with the overall results of the three observers indicate a significant variation in the interpretation of specific biomarkers, contingent upon the observer’s experience. Measuring D(sag) and d(sag) moderately depends on the observer’s experience. Compression of vermis, right cerebellar hemisphere, and pons seems dependent on experience but with considerable uncertainty due to wide CI. Similarly, invasion of right and left superior cerebellar peduncles and medulla oblongata seems dependent on experience, albeit uncertain due to the wide CI. These findings underscore the need for standardising interpretative criteria in the neuroradiographic assessment of cerebellar mutism syndrome to enhance diagnostic consistency and reliability.

In the rapidly evolving field of clinical neuroradiology, the reliance on artificial intelligence (AI) is increasing and becoming inevitable. The study by Sidpra et al. has taken the first essential steps toward integrating AI in cerebellar mutism syndrome prediction [15]. However, the accuracy of these AI algorithms is directly dependent on the quality of their training datasets. The increasing integration of AI in neuroradiology amplifies the risk that biased or inadequate data could lead to erroneous interpretations, underscoring the importance of developing large-scale datasets with reproducible imaging biomarkers to mitigate this fundamental limitation.

Comparing our results with the study by Liu et al. reveals that agreement rates decrease when increasing the number of raters from two to three, particularly for the assessment of compression features [12]. Because of methodological differences between Liu et al. and our current study (study size 10 vs. 20 patients, calculated data with/without CI, the difference in granularity of compression and invasion), we cannot for certain state that there is a significant difference in agreement values when assessing selected ROI among two versus three neuroradiologists. However, based on general statistical considerations, the probability of agreement would decrease with an increasing number of raters. Comparing our results with the study of Sidpra et al., both studies reviewed included imaging biomarkers with three raters [15]. Generally, Sidpra et al. had higher interobserver agreement when evaluating structural changes than our study. It is essential to note the slight difference in chosen imaging biomarkers and how they were reviewed. As with Liu et al., Sidpra et al. reviewed structural changes with a dichotomous approach in contrast to our quantified evaluation. This, again, challenges the comparison with our study (binary vs. ordinal outcome, differences in cohort size, no reported CIs). CIs are not traditionally reported with interrater reliability estimates in studies of interobserver agreement on MRI neuroimaging. To give a more accurate interpretation of the reliability value, we reported the CIs, as we generally believe this is good statistical practice in health research.

Poor interobserver agreement among neuroradiologists can lead to inconsistent diagnoses and treatment plans, including risk assessments, affecting patient outcomes and complicating studies that rely on MRI findings. The study aimed to increase the granularity of the radiological evaluation of structural compression and invasion by introducing a quantitative aspect. This effort may have been overly ambitious, as the results indicated that implementing such an approach was challenging – particularly in the posterior fossa, where densely packed anatomical structures make it difficult to distinguish between normal and pathological boundaries. Upon reviewing the parameters that exhibited a high degree of interobserver variability, it became evident that the discrepancies were due to differences in the quantification of the parameters and rather than the identification of compression or invasion. The variations in observations related to quantifying the degree of severity. The grading of severity was intended to understand the influence of subtle neuroimaging findings on the pathogenesis and severity of cerebellar mutism syndrome. Additionally, the interobserver agreement levels for both ICC and Fleiss’ kappa are based on predefined, arbitrary thresholds. While these classification schemes provide a general framework, they may not fully capture the complexities of interobserver agreement in a clinical setting. Rather than being treated as absolute cutoffs, they should be interpreted as general guidance. We therefore also emphasise the relative differences in agreement across neuroradiographic features, as these variations offer meaningful insights into sources of observer variability.

Our pilot study holds several limitations. For one, the definitions of compression and invasion used in this study were based on experience and consensus of involved experienced neuroradiologists rather than empirical evidence. The definitions are largely consistent with previous literature, facilitating some degree of comparability. Another limitation is the relatively modest number of scans included in the interobserver investigation. Our rationale for piloting the structured report on ten patients was based on assessing feasibility, testing and refining our imaging study protocol, and gathering preliminary insights to ensure a practical and relevant design for a larger study. Furthermore, our choice of reporting CIs introduces uncertainty to our agreement results as the CI depends on the number of observations for ICC and the number of observations and categorical responses for Fleiss’ kappa. Including a larger cohort would diminish the uncertainty of ICC and Fleiss’ kappa, validating our results and ensuring statistical robustness. Additionally, limiting the number of categorical responses would further increase the accuracy of Fleiss’ kappa estimates. Finally, varying scan resolution and available sequences across preoperative diagnostic scans may have influenced the raters’ evaluation, as these factors varied slightly from patient to patient.

Low interobserver agreement has direct clinical implications, particularly in surgical planning, where precise assessment of tumour compression and invasion may be critical for the surgical approach, e.g. balancing the decision on maximal safe resection versus gross total resection. Variability in interpretations could influence surgical decisions regarding resection extent and risk assessment for cerebellar mutism syndrome. Differences in expertise, particularly in rare entities such as paediatric posterior fossa tumours, further underscore the need for standardised reporting frameworks to improve reliability. Our study highlights the need for considering observer-dependent variability by reflecting clinical reality.

## Conclusion

We found reliable agreement among the three observers in tumour size, tumour volume, and Evans index. Invasion of regions of interest generally tended to have higher agreement levels than compression. Our results demonstrate challenges in quantifying imaging biomarkers in the context of cerebellar mutism syndrome due to the complex anatomy of the posterior fossa.

## Supplementary information

## Supplementary Information

Below is the link to the electronic supplementary material.

ESM 1 (PDF 692 KB)

ESM 2 (ZIP 2.81 MB)

ESM 3 (PDF 245 KB)

## Data Availability

Data utilized in this study is part of an ongoing clinical trial with the aim of including 1000 patients. Data from the clinical trial will be available from the corresponding author upon reasonable request when the last included patient has concluded their follow-up in the study.
