# Supplementary material for: Interobserver variability in assessing preoperative imaging biomarkers for cerebellar mutism syndrome: a multiobserver pilot study
Source: Pediatr Radiol. 2025 Jul 17;55(9):1915–26. doi: 10.1007/s00247-025-06326-y (PMC12394353; doi:10.1007/s00247-025-06326-y)
Supplement: Supplementary file 1 — (PDF 692 KB) [file 247_2025_6326_MOESM1_ESM.pdf]

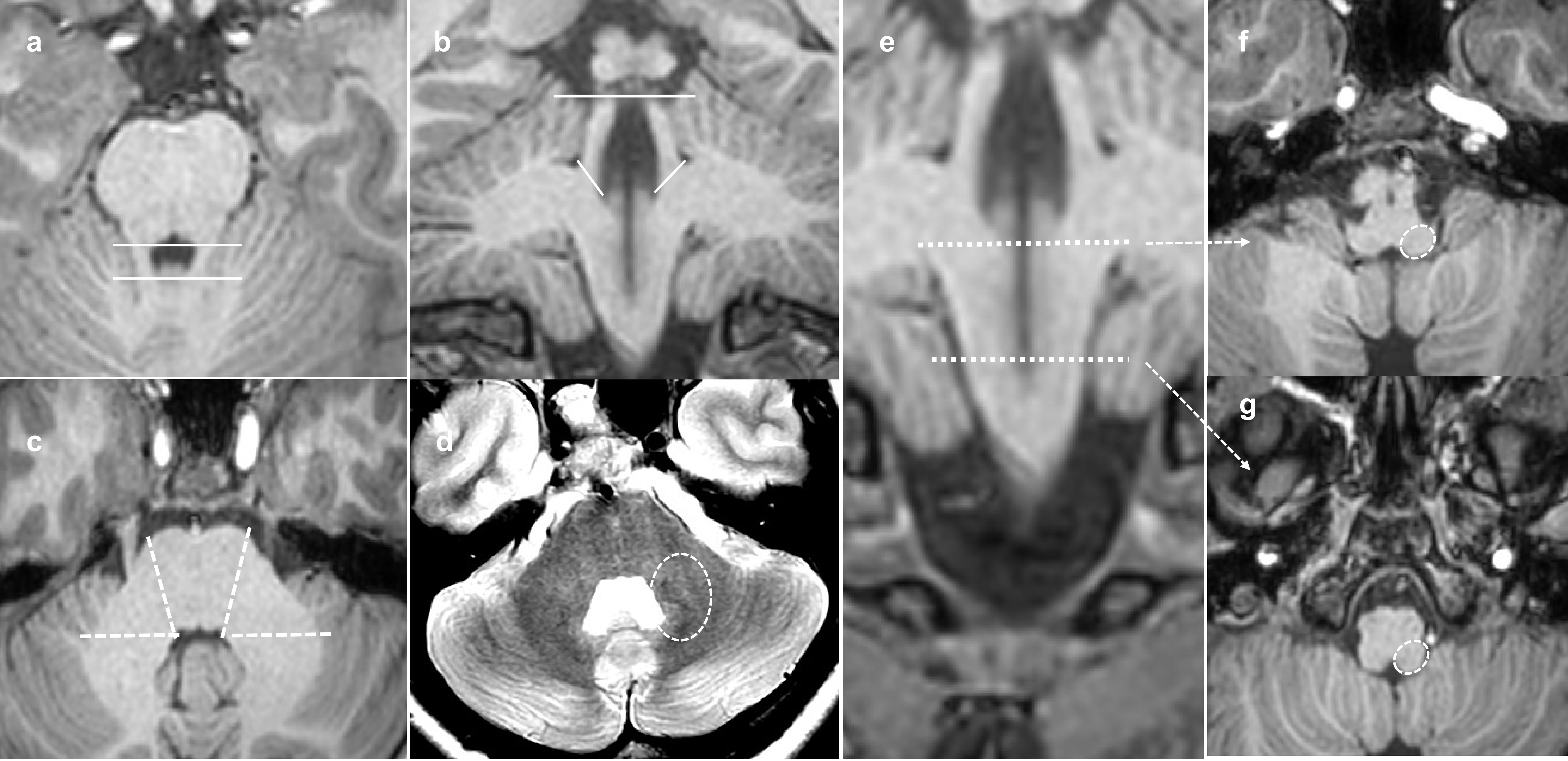


Figure demonstrating the landmarks for the Superior cerebellar peduncle located between the white lines (**a, b**), the middle cerebral peduncles between the dashed lines (**c**), the region of the dentate nucleus within the oval region of interest (**d**) and the inferior cerebellar peduncles between the dotted lines (**e**) with corresponding axial sections (**f, g**)

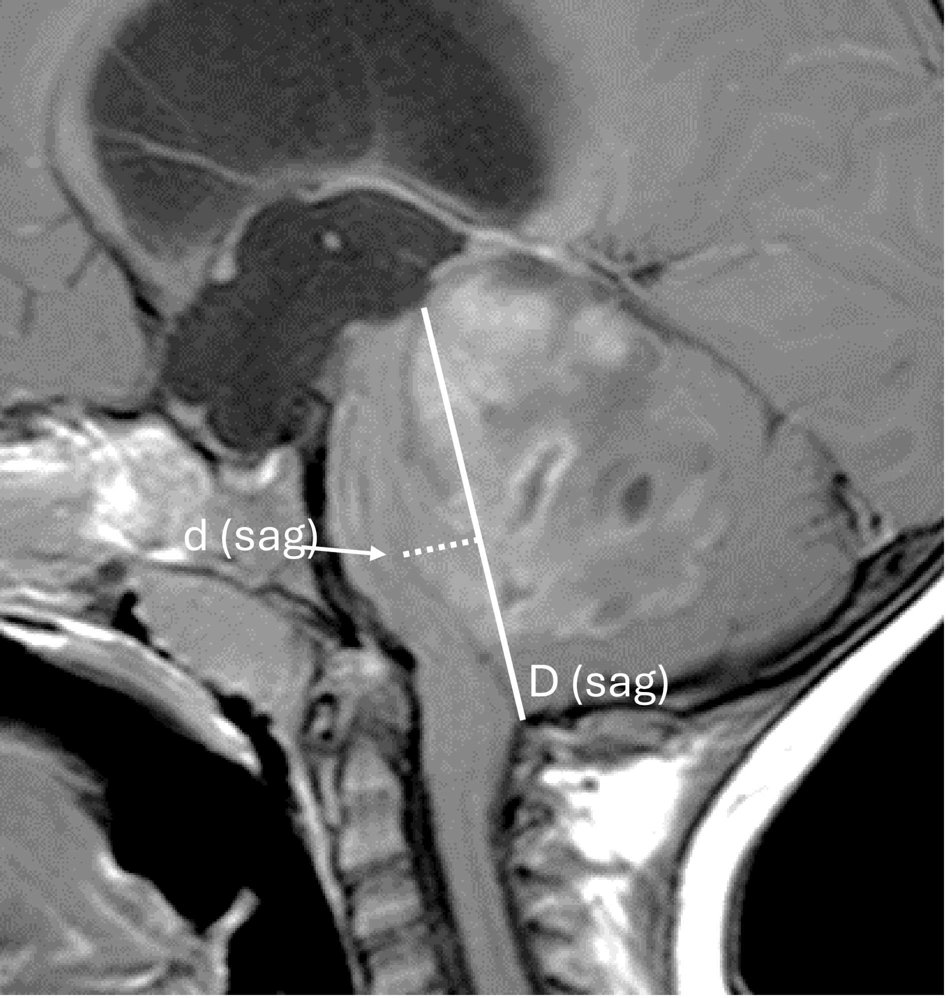


Sagittal T1 post-contrast image demonstrating the landmarks for estimation of D(Sag), the length over which the tumour invades the brainstem, the distance from the upper to lower point of the brainstem invaded by tumour, and d(sag), the depth of invasion of the brainstem.

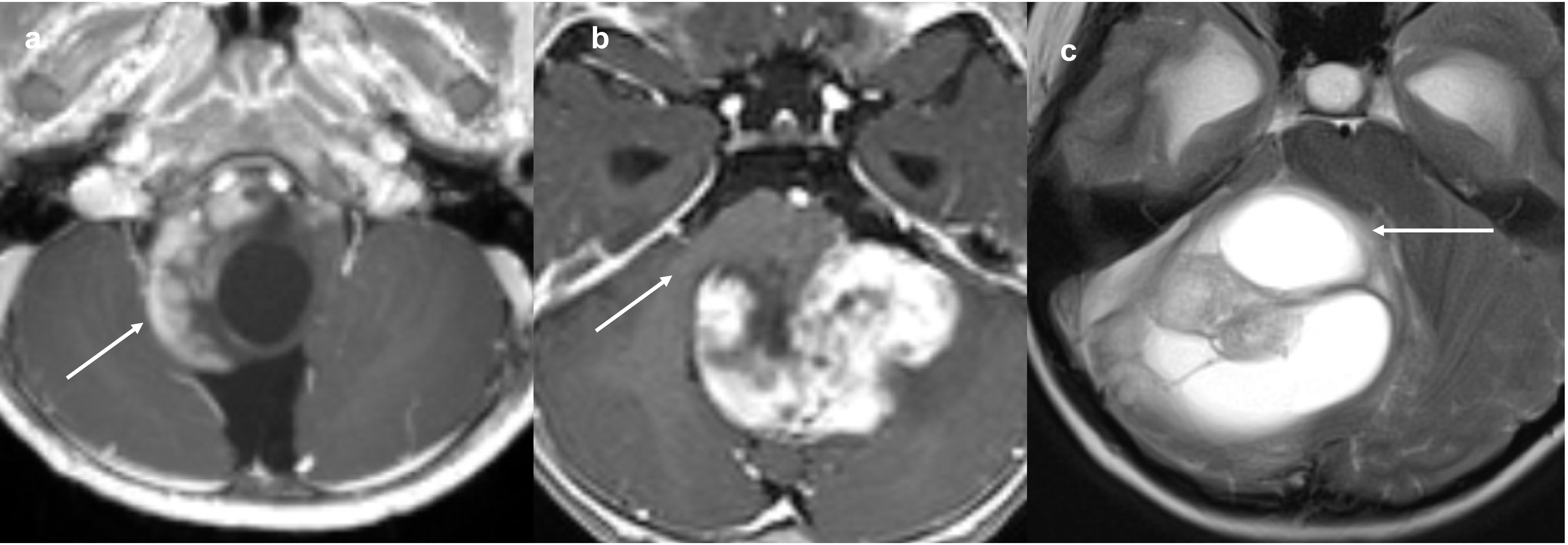


Figure describing the different grades of tumour compression. **a** Score of 2 for  displacement of the right cerebellum without morphological change (*arrow*), **b** Score of 3 for compression of the right MCP, corresponding to displacement and shape change (*arrow*), **c** Score of 4 for moderate to severe compression of the right MCP corresponding to displacement and shape change and distortion of adjacent anatomical structure (*arrow*)

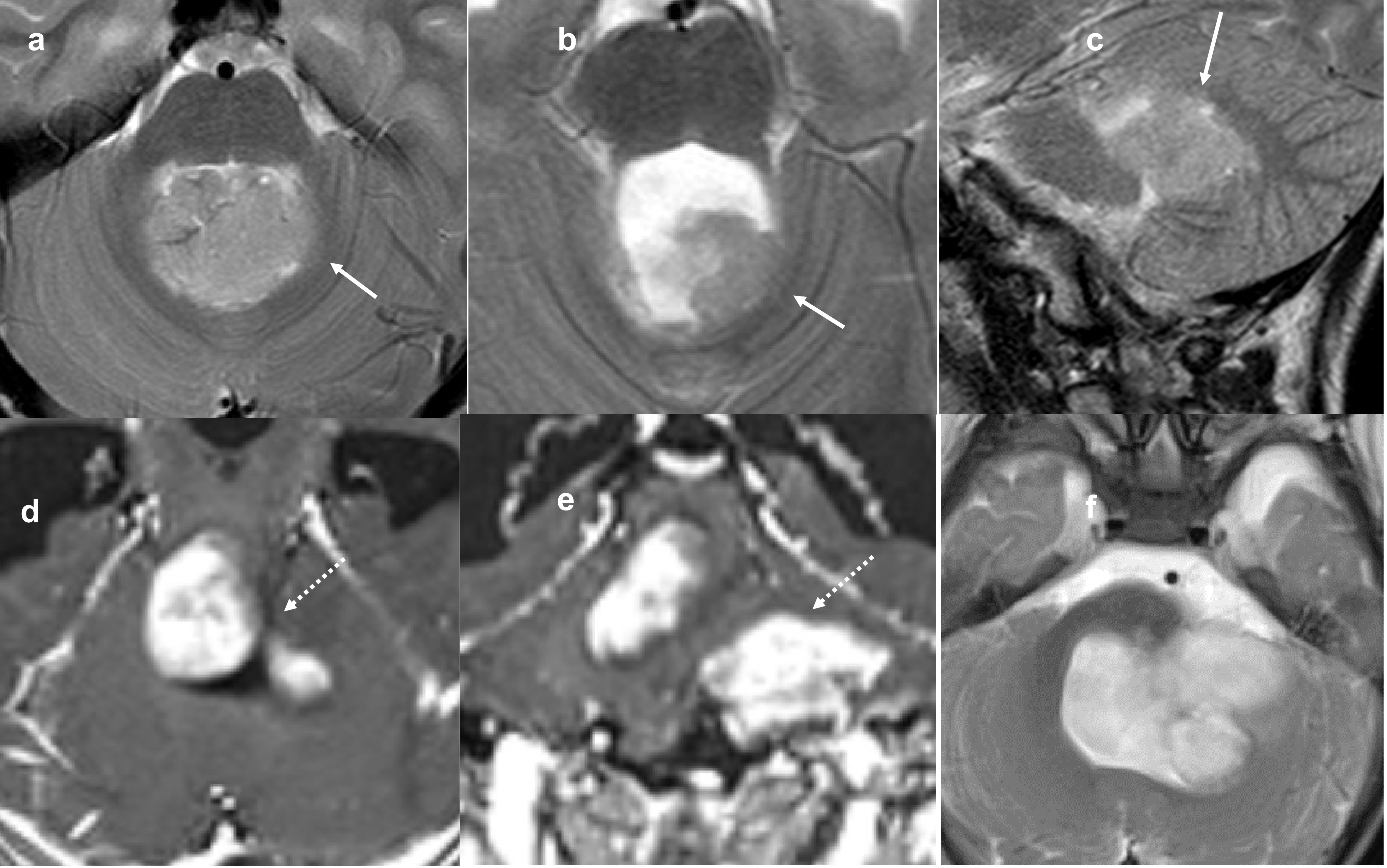


Invasion is characterised by blurring of the margin between the structure and the tumour on 2 or more slices (**a, b,** *arrow*) or in 2 different planes (**a, b, c,** *arrow*). The score of 2 for invasion is provided when there is just blurring of the margin between the tumour and left SCP (**d, e**, *broken arrow*). The score of 3 for invasion of the left MCP, as there is clear penetration by the tumour and loss of MCP visualisation (**f**)

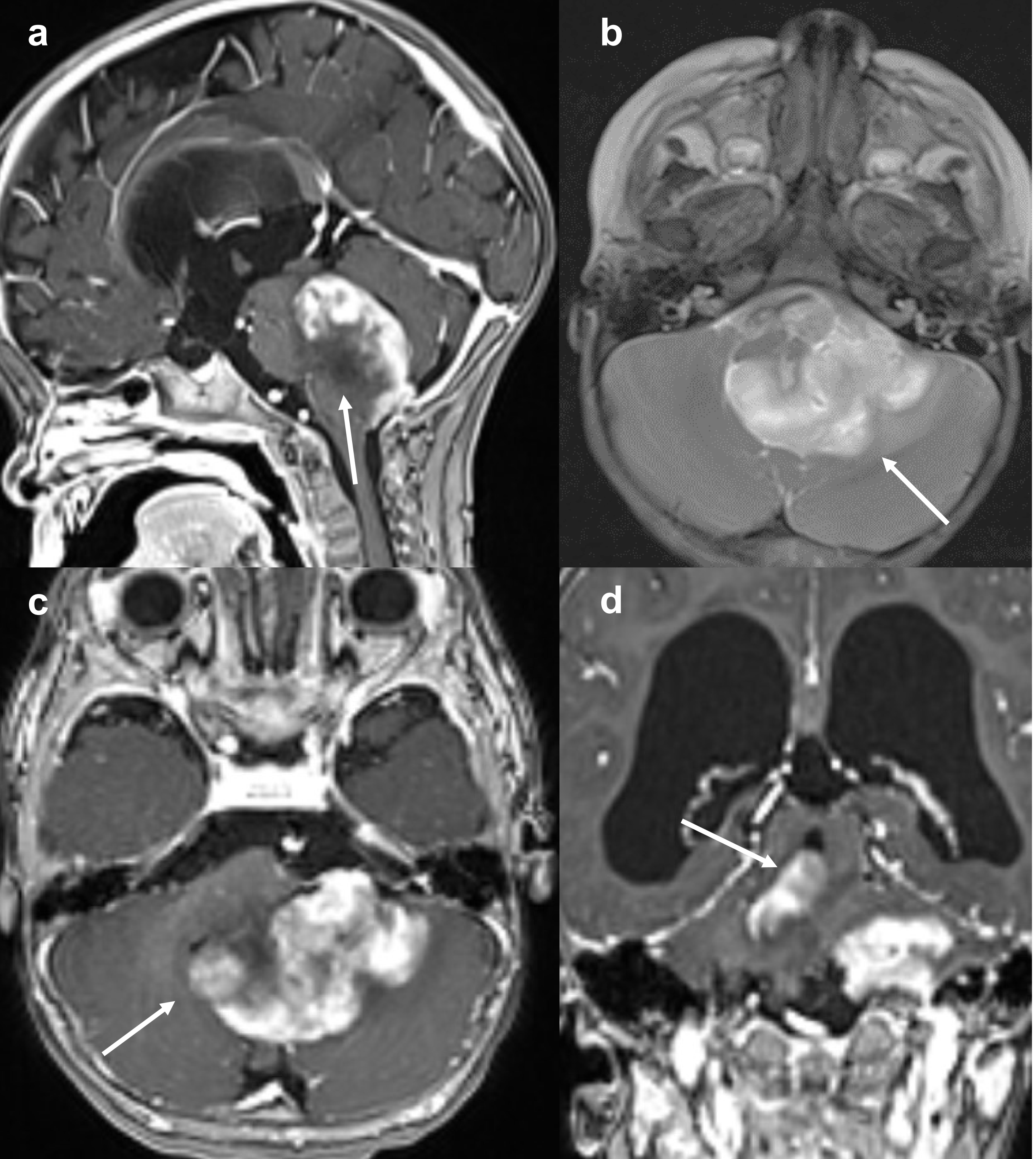


Discrepancies between two observers mainly relating to the grade of compression and invasion. **a** Invasion of the medulla (*arrow*) was rated as 2 (mild) and 3 (moderate to severe), **b** Compression of the left cerebellar hemisphere (*arrow*) was rated as 3 (mild) and 4 (moderate to severe), **c** Compression of the right region of the dentate (*arrow*) was rated as 2 (displacement alone) and 3 (displacement and shape change), **d** Compression of the right SCP (*arrow*) graded as 2 (displacement alone) and 3 (displacement and shape change).
