# Supplementary figures and images for: Interobserver variability in assessing preoperative imaging biomarkers for cerebellar mutism syndrome: a multiobserver pilot study
Source: Pediatr Radiol. 2025 Jul 17;55(9):1915–26. doi: 10.1007/s00247-025-06326-y (PMC12394353; doi:10.1007/s00247-025-06326-y)

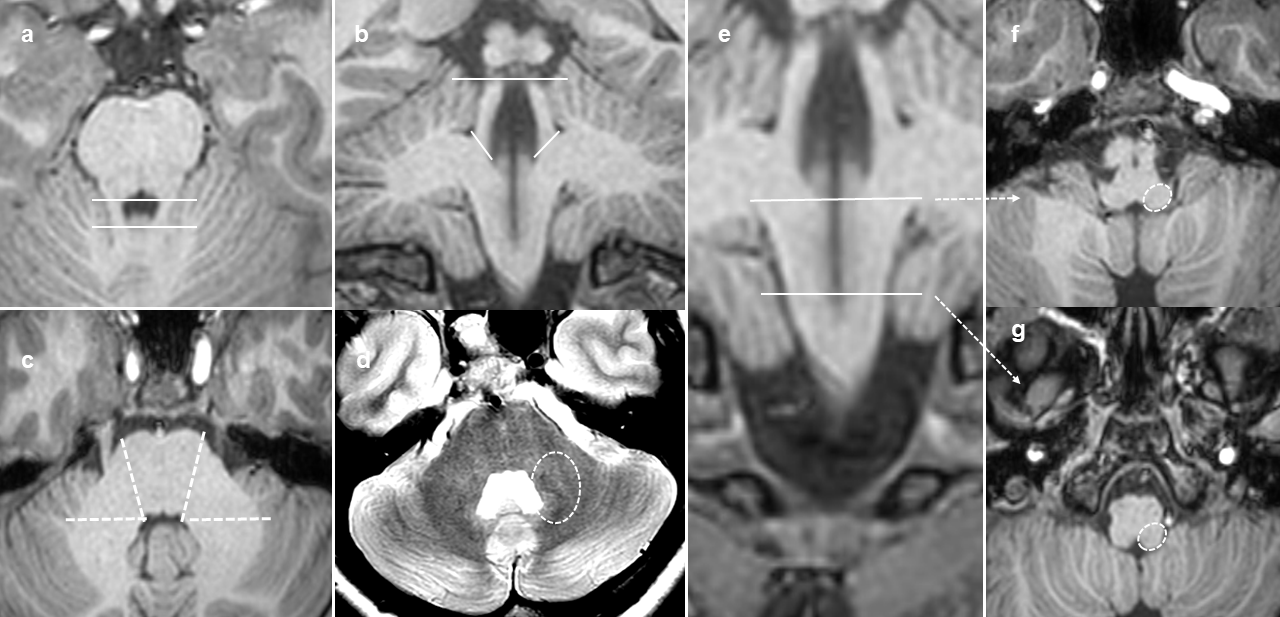

Supplement: Supplementary file 2 — (ZIP 2.81 MB) [file 247_2025_6326_MOESM2_ESM.zip › Slide1_300.TIF]

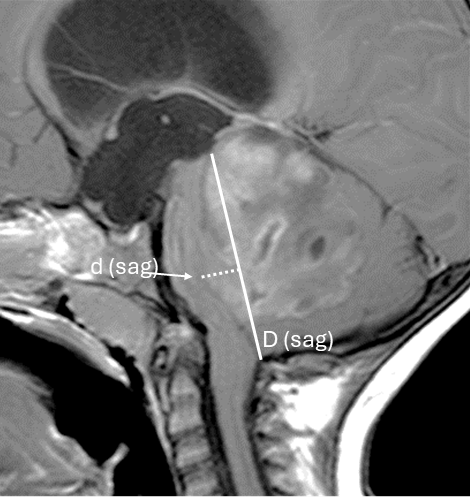

Supplement: Supplementary file 2 — (ZIP 2.81 MB) [file 247_2025_6326_MOESM2_ESM.zip › Slide2_300.TIF]

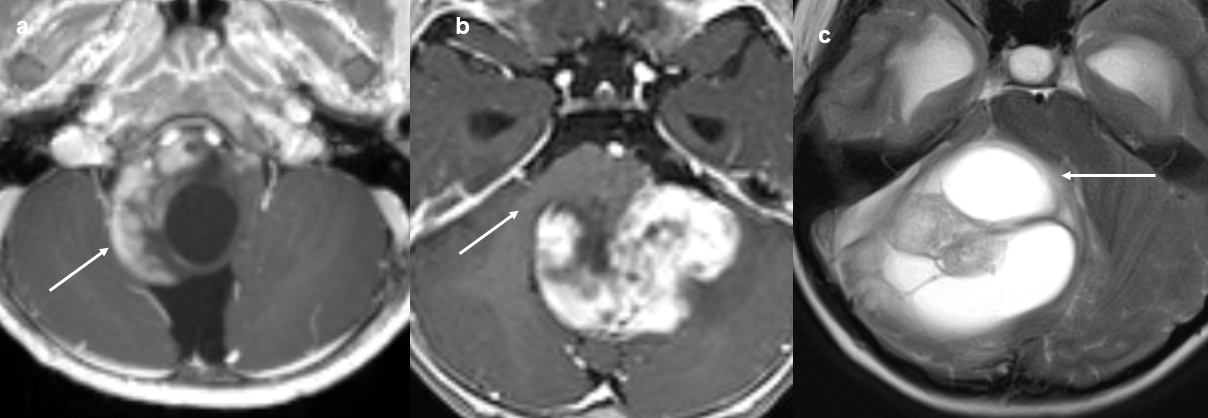

Supplement: Supplementary file 2 — (ZIP 2.81 MB) [file 247_2025_6326_MOESM2_ESM.zip › Slide3_300.TIF]

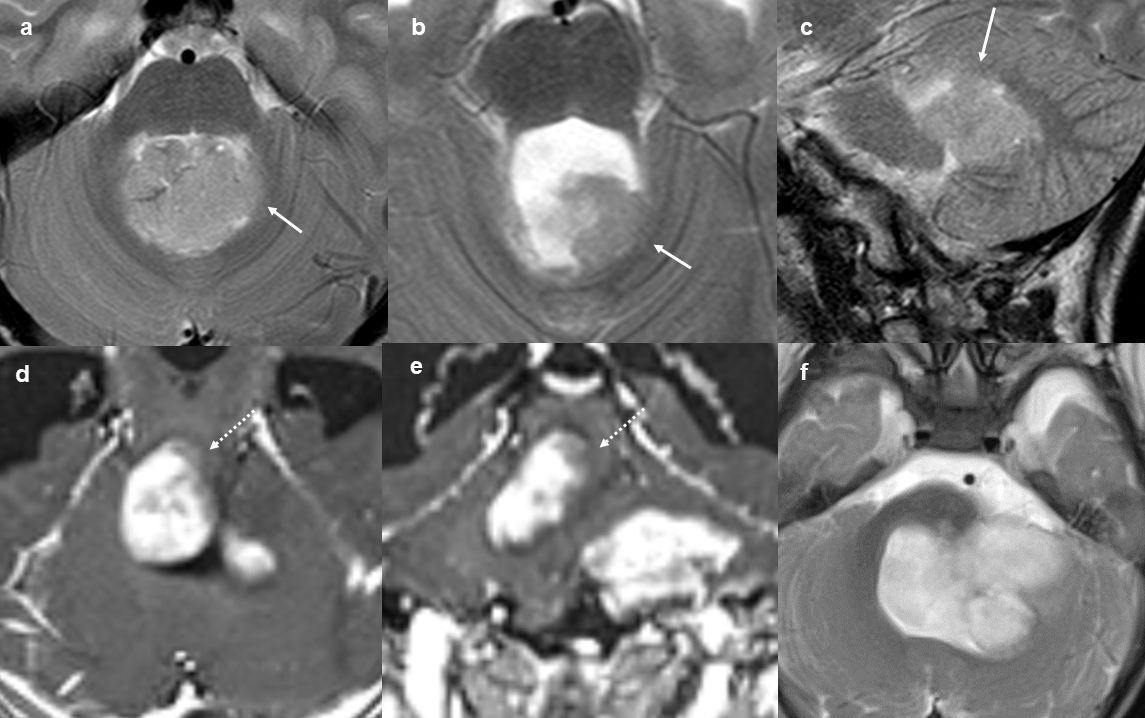

Supplement: Supplementary file 2 — (ZIP 2.81 MB) [file 247_2025_6326_MOESM2_ESM.zip › Slide4_300.TIF]

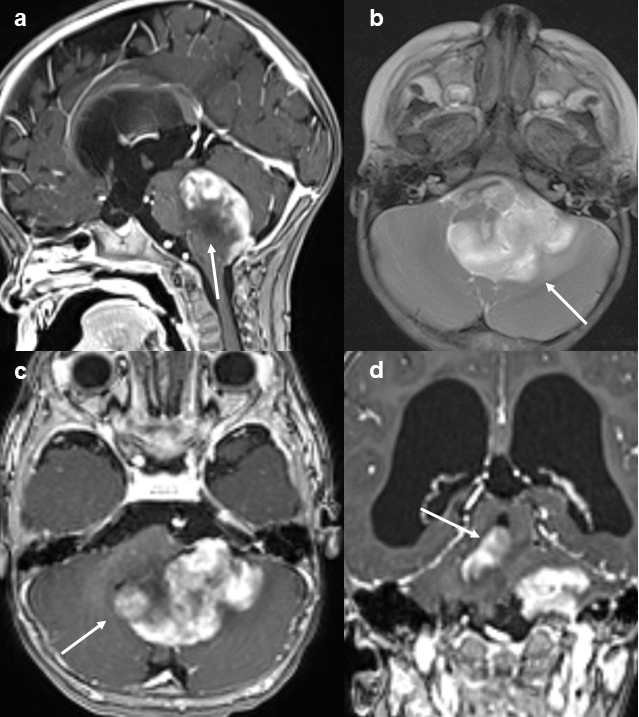

Supplement: Supplementary file 2 — (ZIP 2.81 MB) [file 247_2025_6326_MOESM2_ESM.zip › Slide5_300.TIF]
