# Supplementary material for: Interobserver variability in assessing preoperative imaging biomarkers for cerebellar mutism syndrome: a multiobserver pilot study
Source: Pediatr Radiol. 2025 Jul 17;55(9):1915–26. doi: 10.1007/s00247-025-06326-y (PMC12394353; doi:10.1007/s00247-025-06326-y)
Supplement: Supplementary file 3 — (PDF 245 KB) [file 247_2025_6326_MOESM3_ESM.pdf]

Title: Interobserver variability in assessing preoperative imaging biomarkers for cerebellar mutism syndrome: a multiobserver pilot study

Journal: Pediatric Radiology

Supplementary material

**Supplementary table 1** Consistency for continuous variables	
	ICC consistency (95% CI)
Tumour size	0.95 (0.87;0.97)
Tumour volume	0.95 (0.89;0.97)
Cranio-caudal	0.81 (0.63;0.90)
Anterior-posterior	0.92 (0.84;0.96)
Side-to-side	0.94 (0.88;0.97)
Size of lower brainstem	0.42 (0.07;0.68)
Size of pons	0.61 (0.31;0.79)
Size of Medulla oblongata	0.16 (-0,21;0.49)
D(sag)	0.28 (-0.09;0.59)
d(sag)	0.70 (0.45;0.84)
Evans index	0.90 (0.80;0.95)
Evans A	0.92 (0.83;0.96)
Evans B	0.94 (0.87;0.97)
CI confidence interval, *ICC* Intraclass correlation coefficient	

Interobserver agreement among the 2 most experienced radiologists

**Supplementary table 2** Continuous variables	
	ICC agreement (95% CI)
Tumour size	0.96 (0.91;0.99)
Tumour volume	0.95 (0.87;0.98)
Cranio-caudal size	0.84 (0.63;0.93)
Anterior-posterior	0.96 (0.91;0.99)
Side-to-side	0.98 (0.94;0.99)
Size of lower brainstem	0.56 (0.16;0.80)
Size of pons	0.67 (0.33;0.85)
Size of Medulla oblongata	0.22 (-0.23;0.60)
D(sag)	0.93 (0.84;0.97)
d(sag)	0.92 (0.80;0.97)
Evans index	0.80 (0.56;0.91)
Evans A	0.85 (0.67;0.94)
Evans B	0.83 (0.61;0.93)
CI confidence interval, *ICC* Intraclass correlation coefficient	

**Supplementary table 3** Categorical variables (except invasion and compression)	
	Cohen's kappa (95% CI)
Cystic tumour (>50 % of total tumour size)	0.78 (0.37;1.20)
Haemorrhage in tumour	0.75 (0.34;1.16)
Pathology	0.68 (0.18;1.17)
Location	0.76 (0.19;1.32)
Is the prepontine cistern obliterated?	0.52 (0.11;0.94)
Tumour extension into the aqueduct	1[a]
Signs of intracranial dissemination	1[a]
Signs of spinal dissemination	Not available
Signs of hydrocephalus	0.27 (-0.23;0.76)
Signs of transependymal periventricular oedema	0.78 (0.37;1.20)
Affected nucleus olivaris	0.26 (-0.15;0.67)
[a]No observed disagreement – event did not occur, *CI* confidence interval	

**Supplementary table 4** Invasion and compression of anatomical structure	Cohen's kappa (95% CI)	
	Invasion	Compression
Vermis	0.29 (-0.21;0.78)	0.63 (0.11;1.15)
Hemisphere	0.20 (-0.30;0.70)	0.61 (0.09;1.13)
Right cerebellar hemisphere	0.31 (-0.19;0.81)	0.70 (0.18;1.22)
Left cerebellar hemisphere	0.17 (-0.33;0.66)	0.50 (-0.02;1.02)
Brainstem	0.52 (0.02;1.01)	0.21 (-0.31;0.72)
Mesencephalon	Not available	Not available
Pons	0.51 (0.02;1.01)	0.66 (0.14;1.17)
Medulla oblongata	0.81 (0.32;1.31)	0.22 (-0.30;0.74)
4th ventricle	0.64 (-0.21;1.49)	-0.25 (-0.77;0.27)
Cerebellar peduncles	0.20 (-0.30;0.70)	-0.04 (-0.56;0.48)
Superior cerebellar peduncle	0.44 (-0.05;0.94)	-0.02 (-0.54;0.49)
Right superior cerebellar peduncle	0.62 (0.12;1.11)	-0.05 (-0.56;0.47)
Left superior cerebellar peduncle	0.62 (0.12;1.11)	0.13 (-0.39;0.65)
Middle cerebellar peduncle	0.31 (-0.19;0.81)	0.20 (-0.32;0.71)
Right middle cerebellar peduncle	0.43 (-0.07;0.92)	0.46 (-0.06;0.98)
Left middle cerebellar peduncle	0.44 (-0.05;0.94)	0.22 (-0.30;0.74)
Inferior cerebellar peduncle	0.42 (-0.08;0.92)	0.02 (-0.50;0.54)
Right inferior cerebellar peduncle	0.46 (-0.03;0.96)	-0.01 (-0.53;0.50)
Left inferior cerebellar peduncle	0.46 (-0.05;0.94)	0.26 (-0.26;0.78)
Dentate nucleus	0.22 (-0.27;0.72)	0.01 (-0.50;0.53)
Right region of the dentate nucleus	0.39 (-0.10;0.89)	-0.08 (-0.60;0.44)
Left region of the dentate nucleus	0.40 (-0.10;0.90)	0.17 (-0.35;0.68)
CI confidence interval		
